# Effects of adverse fertility-related factors on mitochondrial DNA in the oocyte: a comprehensive review

**DOI:** 10.1186/s12958-023-01078-6

**Published:** 2023-03-17

**Authors:** Wenying Zhang, Fuju Wu

**Affiliations:** Department of Obstetrics and Gynecology, The Second Hospital of Jilin University, Changchun, Jilin, China

**Keywords:** Mitochondria, mtDNA, Reproduction, Aging, Assisted reproductive technology, Obesity, Diabetes

## Abstract

The decline of oocyte quality has profound impacts on fertilization, implantation, embryonic development, and the genetic quality of future generations. One factor that is often ignored but is involved in the decline of oocyte quality is mitochondrial DNA (mtDNA) abnormalities. Abnormalities in mtDNA affect the energy production of mitochondria, the dynamic balance of the mitochondrial network, and the pathogenesis of mtDNA diseases in offspring. In this review, we have detailed the characteristics of mtDNA in oocytes and the maternal inheritance of mtDNA. Next, we summarized the mtDNA abnormalities in oocytes derived from aging, diabetes, obesity, and assisted reproductive technology (ART) in an attempt to further elucidate the possible mechanisms underlying the decline in oocyte health. Because multiple infertility factors are often involved when an individual is infertile, a comprehensive understanding of the individual effects of each infertility-related factor on mtDNA is necessary. Herein, we consider the influence of infertility-related factors on the mtDNA of the oocyte as a collective perspective for the first time, providing a supplementary angle and reference for multi-directional improvement strategies of oocyte quality in the future. In addition, we highlight the importance of studying ART-derived mitochondrial abnormalities during every ART procedure.

## Background

Oocyte quality affects female fertility and determines subsequent fertilization and embryonic development. The ooplasm provides almost all the nutrients and cellular metabolism sites in the zygote, and the mitochondria play a major role in maintaining the quality of the cytoplasm in the oocyte. Given that mitochondria not only regulate cellular metabolism, but also cell signaling and apoptosis, mitochondrial abnormalities are significant contributors to the decline in oocyte quality.

Mitochondria, also known as the “powerhouses” of the cell, are organelles consisting of two phospholipid bilayer membranes. The inner membrane repeatedly folds inward to form cristae, thereby increasing the surface area to ensure efficient biological reactions [1]. The primary function of mitochondria is to produce adenosine triphosphate (ATP) through oxidative phosphorylation (OXPHOS) via the electron transport chain (ETC). The ETC is located on the inner mitochondrial membrane and is composed of protein complexes I-IV and ATP synthase (complex V). This process releases endogenous reactive oxygen species (ROS) as byproducts that are toxic when they exceed their normal physiological levels. Mitochondria are called “semi-autonomous organelles” because they possess their own genomic mitochondrial DNA (mtDNA), a double-stranded, circular DNA molecule with 16,569 base pairs in humans [2]. mtDNA encodes 22 tRNAs, 13 mitochondrial proteins involved in oxidative respiration, and two rRNAs. Thus, the synthesis of oxidative respiration-related mitochondrial proteins is controlled by both mitochondrial and nuclear genome systems. However, because mtDNA lacks protective histones and repair mechanisms, it is less stable than nuclear DNA and potentially more susceptible to ROS damage. Mitochondrial respiratory dysfunction caused by mtDNA damage further blocks the function of other organelles such as spindle assembly. During spindle assembly, mitochondria are transported to the periphery of the spindle assembly site via the energy-consuming dynein [3, 4]. The process of spindle organization by microtubules and maintaining the dynamic instability of the spindle to help promote chromosome segregation during meiosis and mitosis all require a sufficient ATP supply; otherwise, it will lead to cell cycle arrest and aneuploidy production [5, 6].

**Fig. 1:**
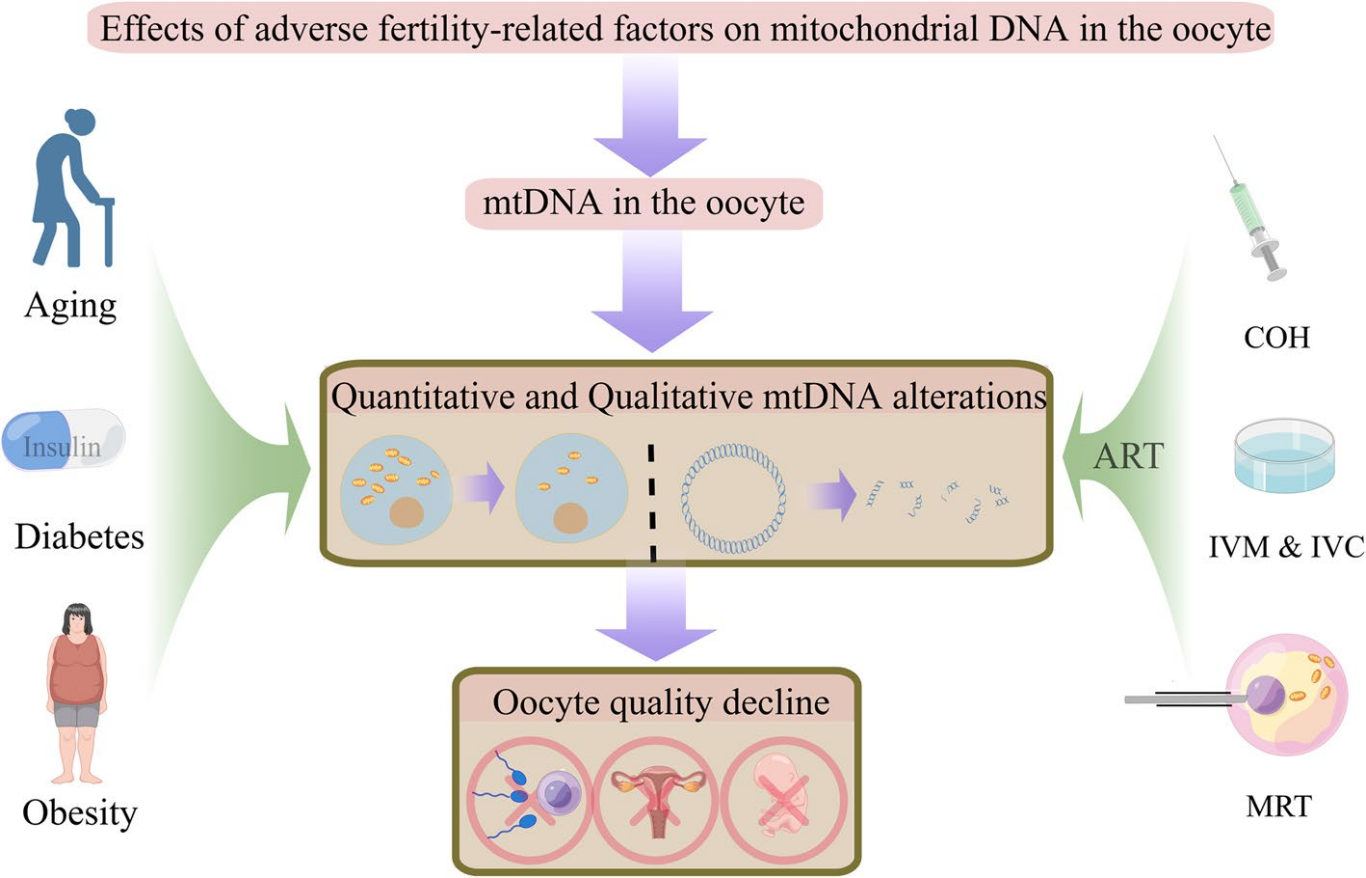
The general idea of this article

Oocytes can suffer from many adverse microenvironment changes that arise from nature (aging), diseases (obesity/diabetes), and assisted reproductive technology (ART). Different oocyte microenvironmental deviations result in different mitochondrial alterations, including mtDNA alterations. The only source of mtDNA in each cell of the offspring is maternally inherited mtDNA, so any abnormalities in the mtDNA in oocytes can cause fertilization and development failure as well as mitochondrial diseases in the offspring. In this review, we have created a collective perspective for the first time that includes a thorough historical analysis of the literature describing mtDNA abnormalities and oocyte quality and have taken into consideration the influence of infertility-related factors on the mtDNA of the oocyte. The collected literature covers 1964 to 2022, and the cited experimental studies cover all existing related reports on humans and animals. Moreover, we demonstrated the intergenerational transmission of mtDNA mutations in oocytes in graphical form, which can be easily understood. Our goal is to provide an overall perspective and reference for using mitochondria-based strategies to improve oocyte quality in the future. There is a pictorial diagram explaining the idea of this article, and it gives readers a brief understanding of the essay before reading the following text (Fig. 1).

To discuss our topic “effects of adverse fertility-related factors on mitochondrial DNA in the oocyte”, we set the first section “mtDNA in the oocyte” to elucidate the characteristics of mtDNA in oocytes and the maternal inheritance of mtDNA. Then we demonstrated the mtDNA alterations in the oocyte by classification of different fertility-related factors including aging, diabetes, obesity, and assisted reproductive technology (ART). ART consist of sub-sections such as controlled ovarian hyperstimulation (COH), in vitro maturation and culture (IVM & IVC), and mitochondria replacement technology (MRT). The mtDNA alterations in the oocyte discussed in this article included both quantitative and qualitative changes, and the quantitative changes can be presented by the number of mitochondria. The qualitative alterations of mtDNA referred to mtDNA mutations, which can impair the normal mtDNA expression. The observed mtDNA alterations contributed to the decline of oocyte quality, which led to subsequent fertilization, implantation, and embryonic development failure.

## mtDNA in the oocyte

In general, each mitochondrion contains more than one mtDNA molecule. The quantity of mitochondria varies widely between individuals and even among oocytes acquired from the same individual. mtDNA replication in oocytes is completed at the germinal vesicle (GV) stage [7]. Estimates of the mtDNA copy number in presumably healthy MII human oocytes range from 2 × 10^4^ to 1.04 × 10^6^ [7–12]. The lower limits of the mtDNA copy number have significant implications for oocyte quality and downstream events such as fertilization, implantation, and embryo development [7, 8, 13, 14]. Reduced mtDNA content may result from defective mitochondrial biogenesis or cytoplasmic maturation [8]. Many studies have demonstrated that there may be a pre-fertilization threshold for mitochondria to guarantee that each blastomere has sufficient copies of mtDNA before mtDNA replication, so that mtDNA is transmitted to all cellular progeny in the post-implantation embryo [14, 15]. mtDNA replication does not occur immediately after fertilization, but oocyte-derived mitochondria are progressively diluted during each round of cell division into daughter cells [16, 17]. Once the “mtDNA set point”, of which the time point has not been clearly defined, is established as the mtDNA replication threshold by the cells of the inner cell mass, mtDNA replication initiates in a differentiation-specific manner [18]. In the female germline, the mtDNA copy number in oocytes during fertilization or preimplantation development can be lowered by an order of magnitude compared to that of post-implantation embryonic development [15]. An advanced blastocyst derived from an oocyte with low mtDNA content is likely compromised through severe mtDNA depletion because limited mitochondria fail to form the networks required for development [14, 19]. However, whether the small amount of mtDNA has an impact on oocyte maturation remains to be confirmed [9, 20].

After fertilization, all of the mitochondria are of uniparental inheritance because the mitochondria from male gametes are eliminated through mitophagy [21–25] or other means [26–28]. As oocyte-derived mitochondria are the only source of the entire mitochondrial complement in offspring, their transmission, replication, and inheritance are strictly regulated. After ovulation, an oocyte with mtDNA alterations can undergo two fates. First, if there are too many mtDNA mutations in the oocyte, fertilization failure occurs. Second, if the oocyte successfully achieves fertilization and cleavage, the mutation level determines the fate of the cells and embryos.

**Fig. 2:**
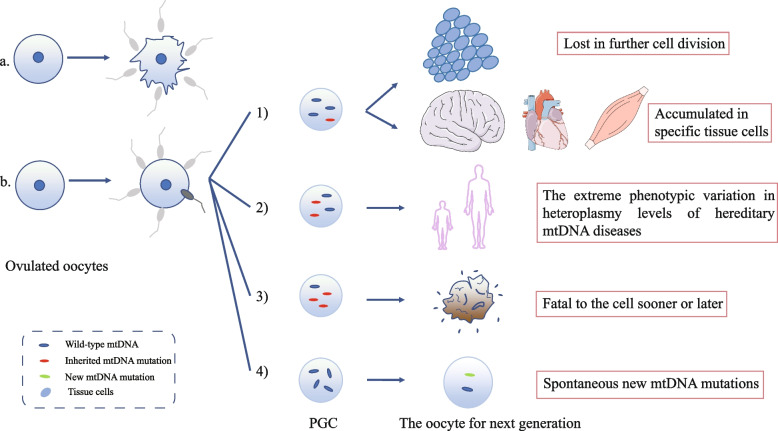
Intergenerational transmission of mtDNA mutations in oocytes

The transmission of mitochondria through generations is hampered by a “bottleneck,” which effectively filters out mtDNA mutations to preserve a homoplasmic (the existence of the same mtDNA haplotypes in an individual) state of mtDNA by limiting its content [12, 29]. Though the definite time of ‘bottleneck’ still lacks direct experimental evidence, the speculation based on population-genetic approaches offers an estimation. Most studies have determined that an effective genetic bottleneck occurs before the formation of mature oocytes [30–32], and the size was calculated to be 1 to 35 mtDNA segregation units in humans [30, 32–34]. However, this bottleneck is not foolproof, at least for mutations with moderate or low frequencies [12, 30, 35]. Genetic drift and mutations may present stronger heteroplasmic (the existence of different mtDNA haplotypes in an individual) effects under the small bottleneck size, compared to the case without the bottleneck. Inherited or de novo variants passing through the bottleneck reach a high-level of heteroplasmy of individual mitochondria by rapid segregation [36]. If the number of point mutations is very low, mutations may be lost in further cell division or accumulate in specific tissues in the offspring after fixation of random genetic drift, leading to deterioration of the OXPHOS system in many age-related diseases. If the number of organelles is sufficiently small, most mutations will be fatal to the organelle or even the cell [37]. During early germ cell development, the transformation from glycolytic to oxidative metabolism exposes harmful mutations to selection to suppress the continuous accumulation of mtDNA mutations, as predicted by Muller’s ratchet for the human population [35, 36]. Thereafter, the complicated processes of oogenesis, folliculogenesis, and follicular competition generate a highly competitive amplification/constraint/mass selection sequence to counteract the ratchet. If there are insufficient winners in the competition between eggs, sterility before oocyte and follicular depletion (endocrine ovarian failure and menopause) should be a physiological choice [38]. Intra-individual Intra-individual selection and transmission of mtDNA molecules through generations is achieved by the Balbiani body (“mitochondrial cloud”) [39–42] which is involved in the bottleneck phenomenon to recruit healthy mitochondria and eliminates dysfunctional ones, letting viable mitochondria duplicate preferentially [39, 43, 44]. Of course, if the phenotypic effect of the mutation is mild or not expressed in the selection environment, mutations can escape the so-called "mitochondrial checkup" [44]. Mutations that escape this mechanism display alterations in the levels of heteroplasmy among one human generation, thus elucidating the extreme phenotypic variations found in human lineages with inherited mtDNA disorders [36]. In addition, harmful mtDNA drift, which means a universal decline in the cytoplasmic health, will also arise in an individual’s offspring, followed by their appearance in the general population, and finally in the entire species [45]. We have used Fig. 2 to summarize the intergenerational transmission of mtDNA mutations in oocytes.

After ovulation, oocytes undergo two fates. a. If there are too many mtDNA mutations in the oocyte, it will directly lead to fertilization failure. b. If they successfully achieve fertilization and cleavage bringing mitochondrial mutations, the mutation level determines the fate of cells and embryos. 1) If the level of point mutations is very low, mutations may be lost in further cell division, or accumulated in specific tissues in the offspring after fixation of random genetic drift, leading to deterioration of the OXPHOS system in many age-related diseases. 2) If the phenotypic effect of mtDNA mutation is slight, or it does not appear in the selection environment, mutations can escape the so-called "mitochondrial checkup," which will show the extreme phenotypic variation in heteroplasmy levels of hereditary mtDNA diseases between generations because of a less stringent mtDNA selection in somatic cells. 3) If the mutation level is high in that it will be fatal to the cell sooner or later. 4) Primordial germ cells (PGCs) contain very few mitochondria, and it is almost impossible to transmit the mtDNA mutation of the previous generation (if carried, they will experience the above process as well). After passing through the “bottleneck” period, they spontaneously produce new mtDNA mutations, which will be passed on to their offspring, and may cause OXPHOS disease in children.

Both nuclear and mitochondrial genetic regulations are required for mtDNA replication, transcription, and expression. Therefore, pathological alterations of mtDNA can be classified into two categories based on their origin: the nucleus and mitochondria. Disorders of nuclear genes involved in the mtDNA process can also lead to mtDNA anomalies, and this type of mtDNA dysfunction is inherited by Mendelian law. Processes influenced by related nuclear genes include oxidative respiration, intergenomic communication, biosynthetic enzymes for lipids or cofactors, and mitochondrial biogenesis [46]. Here, we focused on de novo or maternally inherited primary mtDNA mutations, including point mutations, deletions, insertions, duplications, and rearrangements. Because the “mitochondria bottleneck” occurs early, there is little chance of preserving age-related mutations in early oocytes. Most mtDNA mutations are thought to take place afresh after the “bottleneck” and the earliest development stages of the oocyte or after primordial germ cells (PGCs) migrate to ovaries [29]. mtDNA rearrangements directly cause mtDNA deletion and truncation, which leads to decreased expression of deleted mtDNA and the production of fused gene transcripts [47]. Accumulation of unwanted protein production impairs normal mitochondrial respiratory function, resulting in meiosis and fertilization failure of oocytes due to the inadequate ATP created by OXPHOS. Furthermore, these mitochondria may lose membrane potential and release cytochrome C or other mitochondria-related apoptosis-inducible factors, triggering germ cell apoptosis [44]. Interestingly, the oocyte selection and evolutionary mechanisms were designed to weaken the negative impacts of mtDNA mutations. If oocytes carrying mtDNA mutations possess a large number of wild-type mtDNA copies to guarantee fertilization success, they are likely to enter the initial stage of mtDNA replication. The expected segregation of mtDNA mutations would occur during gastrulation, which is most likely to affect tissues with high ATP demand, such as nervous and muscle tissues. Low-level mutations can be lost during further cell divisions or fixed by random genetic drift, which eventually produce phenotypic results. The appearance of diseases related to mtDNA expression is determined by the proportion of mutant mtDNA to wild-type mtDNA. A changeable threshold for the mutation load depends on the tissue and type of mutation. That is, with age, low-level mtDNA mutations in oocytes may accumulate in particular tissues, leading to the deterioration of OXPHOS in many age-related diseases [48, 49].

## Aging

Aging has a far-reaching impact on oocytes and is the primary factor affecting female fertility. Mammalian models [50, 51] and one human [52] study supported that aging is accompanied by a reduced mtDNA copy number in oocytes, while another human [7] study demonstrated no relationship between the two. Although the link between aging and the mitochondrial content of human oocytes was revealed as early as 1996 [53], the intrinsic reason for contradictory experimental results was not given by Duran et al. [54] until 2011, who stated that reproductive age is a complex variable that may not always coincide with chronological age. They confirmed that reproductive aging may indeed lead to a decrease in the number of mitochondria in human oocytes, and proposed that basal follicle stimulating hormone (FSH) levels and in vitro fertilization (IVF) performance in terms of the total retrieved number of oocytes seem to be more reliable measures than chronologic age in predicting reproductive age. Ovarian aging is characterized by diminished ovarian reserve (DOR), which describes quantitative and qualitative changes in the ovarian oocyte pool. Patients with DOR and ovarian insufficiency also have small mtDNA copy numbers [9, 54], and the number of mitochondria can be estimated using ovarian reserve indicators. The association between basal FSH levels and mitochondrial number may represent an early sign of deteriorating oocyte quality preceding the clinical symptoms of DOR [54]. Similarly, lower mtDNA content has been observed in the polar body of MII oocytes from women of advanced reproductive age (38–45 years old) [55]. A decrease in the mtDNA copy number indicates inadequate redistribution of mitochondria, unsuccessful mitochondrial differentiation, or reduced mitochondrial transcription, contributing to the low cytoplasmic maturation ability of oocytes, poor oocyte fertilization, and compromised embryo development [7, 56]. The discovery of human mtDNA mutations started nearly at the same time as the discovery of the number of mtDNA in oocytes. The first discovery of mtDNA mutations in oocytes were rearranged mitochondrial genomes in 1995, and included the mitochondrial disease-associated 4977 bp “common” deletion [12]. Since then, most human-based studies [57–59] showed no links between mtDNA mutagenesis and aging, but the bovine model [60] led to the proposal that the incidence of deletions increases in an age-dependent fashion. The only human evidence [61] of this correlation indicated that oocytes from older women were more likely to possess the 0.5 kb “common” deletion. We infer that the real root cause is that ovarian aging in laboratory animals corresponds more with the age law. The follicular pool in the ovaries is decisively formed during embryonic life and is no longer renewed. Therefore, when primary oocyte meiosis is blocked at prophase I in primordial follicles, these oocytes undergo a long period of quiescence, during which they may experience an accumulation of mtDNA defects associated with aging. These results indicate that accumulated mtDNA mutations in oocytes may contribute to fertility decline with age and have harmful effects on offspring, such as mitochondrial diseases [62]. Nevertheless, it has been suggested that the number of oocytes containing mtDNA rearrangements decreases significantly with the development of oocytes from the GV to mature MII oocytes in humans, independent of age [58]. Reproductive aging downregulates the expression of respiratory chain genes encoded by mtDNA [63] and impairs mitochondrial biogenesis [50], resulting in a decreased OXPHOS capacity. When the transcriptomes of MII oocytes from young maternal age (YMA) and advanced maternal age (AMA) groups were compared, the most remarkable changes were related to mitochondria. The findings [63] showed that the YMA MII cohort had a higher energy potential, which decreased with age. Ovulation-associated oxidative stress (OS) was the underlying mechanism. Compared with the oocytes of young women, oocytes from women of AMA are more vulnerable to oxidative damage [63, 64] and have a difficult time recovering from oxidative damage [65]. Antioxidant supplementation through oral administration by females before inducing ovulation or through addition to IVM medium of aged oocytes alleviated the above harmful effects, enhancing mtDNA copy number and mitochondrial function, as well as inhibiting the impaired maturation of zygotes, which is often observed in advanced reproductive age [64, 66]. De Boer et al. [52] proposed that a decrease in the number of functional mitochondria with increased maternal age may be due to either point mutation accumulation and/or mtDNA deletions, or the inherent property of recruited oocytes in the later reproductive lifespan. Indeed, fewer mitochondria and a higher rate of common mtDNA deletions in arrested or degenerated oocytes confirmed the hypothesis that these mitochondria may be functionally impaired [54, 56]. Aging oocytes with reduced mtDNA copy numbers can be designed to filter out harmful mtDNA mutations. Only in this way can the female reproductive system ensure that most ovulated oocytes are of good quality, making fertilization, implantation, and embryo development successful. Interestingly, Müller-Höcker et al. [53] observed morphometric-associated increases (in both volume fraction and numerical density) in the mitochondria of mature human oocytes of advanced reproductive age, reflecting subtle changes in OXPHOS capacity, but the changes were not related to mutations in mtDNA or deficiency of respiratory chain enzymes. Due to fewer functional and defective mitochondria (reflected in the reduction and mutation of mtDNA, respectively) in aging oocytes, the energy demands of embryo development offered by the remaining functional mitochondria cannot be met [56]. Mitochondrial replication in older women may increase to compensate for dysfunctional mitochondria because the mechanism of eliminating mutated mitochondria through apoptosis may be damaged [7]. The decreasing tendency and discrepancy of mtDNA deletions from oocytes to embryos could reveal that sporadic mutations accumulate in oocytes during oogenesis and are eliminated by an unknown, perhaps nuclear, mechanism [57]. In addition, a study using a bovine model [60] demonstrated that low-level heteroplasmy in mtDNA was apparent, regardless of the degree of aging or ovarian stimulation. In brief, aging affects the mitochondrial genome, leading to a decrease in the mtDNA content coupled with an increase in age-related mtDNA mutations, which aggravates the effect of mtDNA copy number reduction on oocyte viability, thereby making it difficult for oocytes to recover from mitochondrial dysfunction [65].

## Diabetes and obesity

### Diabetes

Diabetes is a disease characterized by abnormal glucose metabolism. Many studies have reported mitochondrial alterations in various tissues of diabetic models. To investigate the route of adverse effects of maternal diabetes on later generations, results from an established diabetic mouse model demonstrated [67] that there were two routes of maternal diabetes transmitted to the fetus through the oocyte defects: first, nondisjunction caused by meiotic spindle and chromatin defects leading to embryonic aneuploidy; second, mitochondria with abnormal structure and function provide a dysfunctional complement of mitochondria for embryos, which may be spread throughout embryogenesis. Mitochondrial ultrastructure and intracellular distribution, ATP levels, and tricarboxylic acid cycle metabolites were all observed changes in the oocytes of diabetic mothers, accompanied by an increase in the mtDNA copy number. In fact, ATP, ROS, and the pyruvate dehydrogenase complex are related to mitochondrial metabolism, which is needed for correct spindle assembly and chromosome alignment during oocyte meiosis. Qiang Wang et al. [67] suggested that such an increase in mitochondrial biogenesis may be a compensatory phenomenon to ensure adequate generation of ATP in the case of increased requirement or respiratory chain dysfunction, or the increase may be attributed to the decrease in mitochondrial degradation or autophagy [68]. However, biogenesis of the mitochondria promoted by low ATP content or mitochondrial damage leads to the accumulation of low-quality mitochondria in the oocytes, while the inferior mitochondria are partly restored during embryogenesis [69]. Interestingly, oocytes from young diabetic mice were similar to oocytes from a matched aged non-diabetic control group in oocyte aging-related NO-mediated signaling [67, 70].

### Obesity

Obesity has been demonstrated to change the transmission of mitochondria to descendants, whereas the results of mtDNA alterations have been inconsistent. Maternal diet-induced obesity leads to increased mitochondrial potential, mtDNA content, and mitochondrial biogenesis in matured mouse oocytes, which was verified by the upregulation of mitochondrial transcription factor A (TFAM) and nuclear respiratory factor 1 (NRF1) transcripts [71]. In this study, poor mitochondrial metabolism was identified as an OS-mediated dysfunction, which was attributed to the susceptibility of mitochondrial replication to ROS or hormonal or nutritional factors in the obese reproductive environment. Notably, an increased mtDNA phenotype was not observed in the zygotes of obese females [71]. This observation may result from the mtDNA turnover period after fertilization, during which both mtDNA synthesis and degradation occur [72]. This period of mtDNA turnover may provide a mechanism for normalizing abnormal mtDNA levels in the zygotes of obese women. On the other hand, ovulated oocytes from a gene-mutated obese mouse model exhibited normal quantities of mtDNA with reduced mitochondrial membrane potential and a high level of autophagy [73]. In this study, oocytes from obese females formed blastocysts with lower mtDNA levels and heavier fetuses than those from lean mice. They believed that mitochondrial abnormalities were caused by endoplasmic reticulum stress and were reversible during the last phase of oocyte development and maturation. The increase in TFAM and dynamin-related protein 1 (DRP1) amplifies mtDNA replication and mitochondrial fission to enhance oocyte developmental potential. Wu et al. [73] reported that obese mice were unable to amplify DNA during the oocyte to embryo transition, a process known to occur during the implantation preparation stage [15, 16, 74]. Importantly, embryos with low mtDNA levels produce fetal tissues with correspondingly reduced mtDNA content and increased mtDNA sequence variants, reflected by changes in metabolic capacity [75–77], which is consistent with their marked fetal weight gain. Based on the opposing study results, we cannot conclude whether the discrepancy comes from different established models. Further research should be conducted to repeat studies with the same model or to build a uniform model to explore the relationship between mtDNA and obesity.

Supplementation with antioxidants and other substances is a type of adjuvant therapy that can bring mtDNA benefits, especially for populations with metabolic disorders, such as diabetes and obesity. Coenzyme Q10 is a key enzyme in energy production, contributing to the transport of electrons in the mitochondrial respiratory chain, which is supported by the literature for its ability to enhance mitochondrial function by increasing the mitochondrial mass of eggs [78]. No relevant studies have shown that supplementation with other antioxidants and substances, such as resveratrol or glutathione, has an impact on mtDNA.

## ART

ART is a set of clinical treatments and procedures to help infertile women have their own offspring. ART includes artificial insemination (AI), in vitro fertilization, and embryo transfer (IVF-ET). From maternal drug therapy to in vitro manual operation, oocytes can be influenced by controlled ovarian hyperstimulation (COH), freezing, in vitro maturation (IVM), or intracytoplasmic sperm injection (ICSI) and other human interventions. Most investigations on the correlation between ART and mtDNA in oocytes began in 2005, and due to ethical reasons and the limited number of ART cycles, animal findings are far more prevalent than those of humans.

### COH

Repeated ovarian stimulation decreases the mtDNA copy number in mouse oocytes, but this phenomenon can be suppressed by oral administration of L-carnitine, an antioxidant [66]. More importantly, the proportion of mtDNA deletions increased significantly after female injection of exogenous gonadotropin for superovulation in rhesus macaque oocytes [79] and also increased as ovarian stimulation cycles increased in mice [80]. However, a study on golden hamsters demonstrated that excessive doses of gonadotropin and unsuitable hormone combinations would tilt the process of oxidative respiration in the direction of oxidation by increasing the number of mitochondria, thus resulting in deficient intracellular pyruvate and highly produced cytotoxic ROS, producing harmful high levels of ATP [81]. Pyruvate is the sole substrate for the Krebs cycle in mitochondria, and inhibition of effective ATP production may block the in vitro development of mature oocytes after fertilization [81]. Recombinant human growth hormone (rhGH) supplementation before standard ovarian stimulation regimens has been considered as an anti-aging compound to improve ovarian responses in aged women, but mouse evidence suggests that it does not affect mtDNA copy number in oocytes [82].

### In vitro maturation and culture

Because in vivo extracellular conditions cannot be entirely recreated, there are clear variations between in vivo- and in vitro-matured oocytes. Oocytes harvested for IVM showed a decreased mtDNA amount compared to in vivo-matured oocytes. Eggs from preovulatory follicles and matured in vivo, which did not receive in vitro culture procedures, exhibited significantly higher fertilization potentials and blastulation rates in rats [83]. Additionally, the timing of oocyte retrieval is vital. The mtDNA copy number in IVM oocytes increases linearly with the diameter of the antral follicles when retrieving eggs [83]. Although oocytes from small antral follicles can achieve IVM, their mtDNA number, ATP content, and proportion of oocytes with peripherally distributed mitochondria are markedly lower than those from preovulatory follicles and in vivo-matured oocytes [83]. Some clinical IVF and ICSI failures may result from low mtDNA and ATP levels, which may prevent meiotic birefringent spindle formation in human in vitro-matured oocytes [8, 84]. Vitrification sets the relative expression of mtDNA-coded ATP6 and ATP8 as the opposite model to that of fresh bovine oocytes [85].

Data from non-human primate model rhesus monkeys showed that suboptimal medium conditions may directly perturb mitochondrial gene expression, as well as the nuclear components that govern mitochondrial biogenesis [86]. By interfering with the expression of sirtuin 3 (SIRT3), which is a nuclear encoded mitochondrial NAD + -dependent deacetylase, human IVM oocytes showed a considerable reduction in the number of mitochondria, expressed as mitochondrial biogenesis deficiency, which influences the developmental efficiency from oocytes to blastocysts [87]. Deficiencies in regulating nuclear–mitochondrial crosstalk and the susceptibility of mitochondrial biogenesis to in vitro maturation and culture may underlie the poor success rates of human ART. In particular, continued development and maturation of the oocyte within the ovarian follicle in vivo facilitates the production of the highest developmental potential oocytes, which are defined by obtaining sufficient ATP production and the minimum mitochondrial threshold required for continued development [86, 88]. Furthermore, an experiment in equines with reproductive aging showed that IVM led to the general downregulation of genes participating in the replication and function of mitochondria via action on mitochondrial replication, repair, or ability to resist the effects of ROS, irrespective of maternal age [89]. Compared with oocytes from natural cycles, human oocytes that only received COH or IVM intervention revealed that both had reduced mtDNA, but different clinical procedures led to malignant outcomes, low IVF efficiency, and high embryonic loss rate through different ooplasmic alterations [90]. Oocytes derived from the COH procedure had reduced mitochondrial membrane potential, ATP production, and percentage of normal cytoskeletons, whereas oocytes derived from the IVM procedure had a lower percentage of normal cytoskeletons but higher ROS production. Interestingly, a recent study found that in vitro mouse oocytes had increased mtDNA copy number to compensate for decreased mitochondrial function [91]. A previous bovine study [92] may offer an explanation; the final stage of follicular development showed a remarkable increase in mtDNA replication in competent oocytes, which is linked to enhanced anaerobic glycolysis and pentose phosphate pathway (PPP) activities after the oocytes have reached their final size. Therefore, it is difficult to distinguish fully developed oocytes based on morphology in clinical practice, which is a potential reason for the low development rate of IVM oocytes. KEGG enrichment and protein–protein interaction (PPI) network analysis [93] of in vivo and in vitro matured oocytes from mice of advanced reproductive age showed that OXPHOS was the most significantly enriched and interactive pathway. The majority of the hub genes were mtDNA-encoded subunits of respiratory chain complex I. Pretreatment with necrostatin 1 during IVM might be beneficial to improve the developmental competence of mouse oocytes, and the mitochondrial genome might be potentially downregulated at the gene level [94]. Adding melatonin, a universal anti-aging agent, into IVM medium to alleviate oocyte oxidative stress and determine whether it affects the amount of mtDNA, showed contradictory results in different species [95, 96]. Park et al. have suggested that mitochondrial apoptosis can be reduced to sustain mtDNA function [97].

### Current situation of mitochondria replacement technology (MRT) as a new ART to rescue oocytes’ mitochondrial abnormalities

To date, although there have been many reports on the treatment of infertility by improving the mitochondrial function of oocytes, the strategies targeted to mtDNA are very limited, and most of them were developed from mitochondrial replacement technology (MRT), which originally aimed to eliminate the transmission of maternally inherited mtDNA diseases in the next generation. MRT involves replacing some or all of the cytoplasm of patients’ germ cells so that the patient’s nucleus and its genetic materials are surrounded by healthy mitochondria and cytoplasm, thereby developing a healthy baby that is free from the effects of poor-quality mitochondria. According to the different operation modes, MRT can be divided into germinal vesicle transfer (GVT), polar body transfer (PBT), maternal spindle transfer (MST), and pronuclear transfer (PNT). Research on the application of MRT to improve reproductive outcomes in infertile patients showed that it only provides benefits for the aging population [98] and the population with fertilization failure after ICSI [99]. Furthermore, only mitochondria from stem cells or young donor germ cells should be used in the reproductively aging population, as mitochondrial abnormalities in these two types of cells are relatively rare. It is worth noting that regardless of the type of MRT, patient’s mtDNA will be inevitably carried into the newly organized oocyte when transferring patient’s oocyte contents. There are two problems in new cells with small amounts of patients’ mtDNA. First, it may be incompatible with nuclear DNA, creating a cross-talk barrier between the cytoplasm and nucleus. Second, the low level of mitochondrial genome heteroplasmy may alter mtDNA genotype stability, affecting the continued elimination of patient’s mtDNA, ultimately leading to the accumulation of mtDNA mutations in differentiated tissues of future generations through genetic drift [100]. Even if a normal mtDNA genotype is carried in the reorganized oocyte, mitochondrial function can be altered when two normal genotypes coexist at approximately equal levels [101]. Given this situation, MRT should be combined with preimplantation genetic diagnosis and prenatal screening.

Another category of treatment is the injection of normal mitochondria or mtDNA into the oocytes of patients, which evidently reverses the function of inferior mitochondria by increasing the proportion of normal mitochondria. Autologous cells, such as ovarian cells, oogonia stem cells, oocytes, stromal cell lines, hematopoietic stem cells, and adipose-derived stem cells [98], are safer sources of avoiding heterologous mitochondria. Owing to unknown side effects and ethical problems, the United States Congress has prohibited the Food and Drug Administration (FDA) from accepting applications for clinical research using MRT since December 2015. Therefore, clinical research using MRT in humans cannot proceed legally in the United States. Even in the United Kingdom, where mitochondrial donation was first legalized, MRT is only used to prevent the transmission of mitochondrial diseases and not to treat infertility.

## Conclusion and future directions

Since the current review on the mitochondrial status of oocytes from infertile patients focuses on mitochondrial function and ignores the role of genetic material, our review details the impact of reproductive-related infertility factors on the quantity and quality of mtDNA in oocytes. A summary is provided in Table 1. In many cases, a combination of multiple infertility factors cause infertility in an individual. A detailed understanding of the effect of each infertility-related factor on mtDNA is conducive to the clinical development of strategies for simultaneous treatments to improve fertility. Because mtDNA is positively correlated with the number of mitochondria in cells, abnormalities in mtDNA disrupt the balance of mitochondrial dynamics. Our paper provides a literature summary of the current research hotspots including the mitochondrial dynamics of oocytes, and provides a reference for the use of mtDNA as a biomarker or indicator of impaired mitochondrial function in preimplantation genetic diagnosis. MRT has a limited clinical application for the treatment of mtDNA abnormalities caused by infertility. Due to ethics and a series of unknown problems in MRT, we should acknowledge the promises and highlight the pitfalls. Furthermore, although ART can rescue some poor maternal physical conditions, MRT cannot avoid mitochondrial impairment from ART. Therefore, it is more feasible to delve into how to prevent mtDNA damage from ART. Newly developed mitochondrial genome editing is a more targeted method for the treatment of adverse pregnancy outcomes caused by mtDNA mutations. Transcription activator-like effector nucleases (mitoTaLens) [102] or zinc finger nucleases (mtZFNs) [103] are localized to the mitochondria and degrade different types of pathogenic mtDNA mutations to achieve permanent elimination of pathogenic mutations via cleavage. Mitochondrial-based CRISPR/Cas9 gene-editing technology appears to offer an alternative to MRT for greater progress in infertility therapeutics. It will take decades to understand the mechanism-based benefits and risks of gene editing technology through long-term follow-up of the limited applications of this technology on mitochondrial diseases. Only after sufficient preventive measures can be conducted by scientists and doctors will it be the right time to carry out gene editing in the human germline.

**Table 1:** Literature collection of mitochondrial DNA alterations in different adverse fertility-related factors

Mitochondrial DNA (mtDNA) alterations	Aging	Assisted reproductive technology (ART)			Diabetes	Obesity
		Controlled ovarian hyperstimulation (COH)	In vitro maturation and culture	Mitochondria replacement technology (MRT)		
Quantitative alterations, including the changes of mitochondria number	Human [7, 9, 52–56, 64] Mice [50, 51, 66, 82] Bovine [65]	Human [90] Mice [66, 82] Golden hamsters [81]	Human [8, 84, 90] Mice [91] Rats [83] Equine [89]		Mice [67]	Mice [71, 73]
Qualitative alterations: mtDNA mutations (point mutations, deletions, insertions, duplications and rearrangements) and the expression of mtDNA	Human [12, 56–59, 61, 63] Bovine [60]	Mice [80] Rhesus macaques [79]	Mice [93] Rhesus macaques [86] Bovine [85]	Human [100]		

The blank space indicates the absence of related literature

## Data Availability

The datasets generated and/or analyzed during the current study are available in the MEDLINE repository. https://pubmed.ncbi.nlm.nih.gov/ Figure 1 was drawn by Figdraw. https://www.figdraw.com/static/index.html.

## Abbreviations

mtDNA: Mitochondria DNA
ART: Assisted reproductive technology
ATP: Adenosine triphosphate
OXPHOS: Oxidative phosphorylation
ETC: Electron transport chain
ROS: Reactive oxygen species
GV: Germinal vesicle
PGCs: Primordial germ cells
FSH: Follicle stimulating hormone
IVF: In vitro fertilization
DOR: Diminished ovarian reserve
YMA: Young maternal age
AMA: Advanced maternal age
OS: Oxidative stress
AI: Artificial insemination
IVF-ET: In vitro fertilization and embryo transfer
COH: Controlled ovarian hyperstimulation
IVM: In vitro maturation
ICSI: Intracytoplasmic sperm injection
rhGH: Recombinant human growth hormone
SIRT3: Nuclear encoded mitochondrial NAD + -dependent deacetylase
KEGG: Kyoto Encyclopedia of Genes and Genomes
PPI: Protein–protein interaction
TFAM: Transcription factor A, mitochondrial
NRF1: Nuclear respiratory factor 1
DRP1: Dynamin-related protein 1
MRT: Mitochondria replacement technology
GVT: Germinal vesicle transfer
PBT: Polar body transfer
MST: Maternal spindle transfer
PNT: Pronuclear transfer
